# Microbiological effect of topically applied *Weissella cibaria* on equine pastern dermatitis

**DOI:** 10.3389/fvets.2024.1493756

**Published:** 2025-01-06

**Authors:** Eva Styková, Igor Valocký, Jana Kačírová, Lívia Kolesár Fecskeová

**Affiliations:** ^1^University Veterinary Hospital, Clinic for Horses, University of Veterinary Medicine and Pharmacy in Košice, Košice, Slovakia; ^2^Institute of Plant Genetics and Biotechnology, Plant Science and Biodiversity Centre, Slovak Academy of Sciences, Nitra, Slovakia; ^3^Associated Tissue Bank, Faculty of Medicine, Pavol Jozef Šafárik University in Košice, Košice, Slovakia

**Keywords:** equine pastern dermatitis, horse, microbiota, skin, *Weissella cibaria*

## Abstract

Equine pastern dermatitis (EPD) is a multifactorial disease with a change in the skin microbiome. The present study monitored the influence of *Weissella cibaria* Biocenol™ 4/8 D37 CCM 9015 stabilized on alginite on the skin microbiota of healthy horses and model patients with EPD. Based on clinical signs, EPD lesions were identified as exudative or proliferative forms. A comparison of the initial microbial community based on 16S rRNA amplicon sequencing revealed that there was a statistically significant difference between healthy vs. exudative (*R* = 0.52, *p* = 0.003) and exudative vs. proliferative communities (*R* = 0.78, *p* = 0.043). The healthy skin microbiota was dominated by the families Corynebacteriaceae (19.7 ± 15.8%) and Staphylococcaceae (15.8 ± 10.7%). *Streptococcus* (11.7 ± 4.1%) was the dominant genus in the exudative group together with *Corynebacterium* (11.0 ± 3.8%), while *Staphylococcus* (15.6 ± 14.5%) dominated the proliferative group. The genus *Staphylococcus* represented only 0.5% of the exudative skin microbial community, a major difference between EPD-affected lesion types. Upon application, there was a statistically significant shift in community composition in all the groups, including the healthy community; however, the change was the most significant in the exudative community. On average, the genus *Weissella* represented 80.0 ± 13.3% of the exudative and 49.0 ± 30.0% of the proliferative bacterial community during treatment. One week after the application period, richness and diversity increased and were comparable in all groups. The application of the *W. cibaria* strain was associated with a significant decrease of the genera *Staphylococcu*s, *Moraxella,* and *Rothia* in the proliferative group and with a decrease of *Streptococcus* and *Clostridium* in both exudative and proliferative groups. Based on our results, we conclude that a topically applied *W. cibaria* RIF^R^, stabilized on alginit, induced potentially beneficial shifts in the composition of the skin microbiota.

## 1 Introduction

Equine pastern dermatitis (EPD) is a syndrome manifesting as a non-specific cutaneous reaction pattern on the distal extremities (1). In general, it can be said that it is a multifactorial disease with a change in the skin microbiome. Clinical manifestations are characterized by skin lesions located on the palmar/plantar side of the pastern and often extend dorsally and proximally to the pastern and fetlock. Painful stretching of the affected skin is evident when the horse moves and can lead to lameness (2). In the mild form of EPD alopecia, dry scales and crusts are present. Thickening of the skin, pruritus, and pain are variable. The exudative form of EPD is characterized by erythema, erosion, alopecia, and serous to purulent crusting dermatitis, often accompanied by epidermolysis and vasculitis. The chronic proliferative form is characterized by excessive granulation tissue that becomes cornified, along with nodular proliferations of hyperkeratosis, and lichenification. Fissures and papillomatous areas may develop (3). Mild to moderate (exudative) manifestations of EPD predominate in lighter breeds, while progression to severe debilitating forms (chronic proliferative form: verrucous dermatitis or “grapes”), including chronic progressive lymphedema, is observed in draft horses (1). In draft breeds, associations with *Chorioptes* infestation have been largely reported (3–5), but not associated with lesion severity (6). *Dermatophilus congolensis*, which was previously mentioned as a significant factor (7), was identified scarcely or not at all (5). Fungal infections are not associated with EPD (8).

The most common cause of EPD is staying in a humid environment. Moist skin loses its protective barrier against the invasion of opportunistic pathogens (3). In the treatment, general/local application of antibiotics is used, but in addition to the risk of inducing resistance, it also has a negative effect on the skin microbiota. Restoration of bacterial homeostasis is considered essential in wound healing, and the appropriateness and management of antibacterial therapy and alternative treatment options are still debated (9, 10).

The probability of EPD recurrence is high. Despite its high incidence, the etiology and pathogenesis of this disease remain poorly understood (11, 12). The current method of microbiota analysis associated with EPD in clinical practice is bacteriological culture. An alternative method that is becoming increasingly popular in human and veterinary medicine is next-generation sequencing (NGS) of the bacterial 16S rRNA gene (13). This method revealed that pasterns affected by EPD showed reduced diversity in the microbiota (12).

The study aimed to monitor the influence of the beneficial strain *Weissella cibaria* Biocenol™ 4/8 D37 CCM 9015 stabilized on alginite on the composition of the skin microbiota of healthy horses and model patients with EPD by 16S rRNA amplicon sequencing.

## 2 Materials and methods

### 2.1 Preparation of *Weissella cibaria* Biocenol™ 4/8 D37 CCM 9015 RIF^R^

*Weissella cibaria* was sampled from healthy equine skin and identified as described in Styková et al. (14). The strain was deposited in the Czech Culture Collection (Masaryk University, Brno, Czech Republic) as *W. cibaria* Biocenol™ 4/8 D37 CCM 9015. The strain was preserved in Microbank™ (Pro-Lab Diagnostics, Richmond Hill, ON, Canada) at −70°C. A spontaneous rifampicin-resistant mutant of the *W. cibaria* strain was isolated by inoculation of a night culture of the strain onto MRS agar (Carl Roth GmbH + Co., KG, Karlsruhe, Germany) containing 30 μg/mL rifampicin, followed by 10 serial passages (Sigma Chemical Co., Poole, UK). The obtained resistant colonies were incubated anaerobically on the abovementioned plates (BBL GasPak™ Plus, BD, Franklin Lakes, NJ, USA) at 37°C for 48 h. By this procedure, a resistant form of strain *W. cibaria* Biocenol™ 4/8 D37 CCM 9015 RIF^R^ (*W. cibaria* RIF^R^) was obtained.

### 2.2 Fossile carrier

Pre-dried, ground alginite (Algiwo, s.r.o., Lučenec, Slovakia) with a grain size of 0.1 m–1 mm, subjected to gamma-irradiation (Bioster, Veverská Bítýška, Czech Republic), was used as a carrier.

### 2.3 Inoculation of alginite with *Weissella cibaria* RIF^R^

Individual colonies grown on MRS agar containing 30 μg/mL rifampicin were resuspended in 5 mL of sterile saline solution (Oxoid, England) to obtain a density corresponding to 5 McFarland (approximately 1.5 × 10^9^ colony forming units (CFUs)/mL). The suspension with a volume of 5 mL was added to 95 mL of MRS broth (Oxoid, Basingstoke, UK) and incubated in a water bath (LSB Aqua Pro, Grant Instruments, Cambridge, UK) with shaking at 110 rpm and 37°C for 18 h. Then, each sample of radiation-sterilized alginite (100 g) was inoculated with 83 mL of grown *W. cibaria* RIF^R^ strain. Paste consistency was obtained. Inoculated alginite was stored at room temperature until application.

### 2.4 Numbers of *Weissella cibaria* RIF^R^ in alginite

The numbers of *W. cibaria* RIF^R^ were determined in the alginite samples at application time for the first and second weeks of application. Samples weighing 0.5 g were taken from the inoculated alginite (100 g) in three places. Decimal diluted samples (0.1 mL of dilutions from 10^−3^ to 10^−6^) were inoculated onto MRS agar containing 30 μg/mL of rifampicin. The plates were incubated anaerobically at 37°C for 48 h and the counts of *W. cibaria* RIF^R^ in triplicates were determined.

### 2.5 Animals and application of *Weissella cibaria* RIF^R^ in alginite

Six healthy mares of the Norik breed Muráň Plain type and 12 mares of the same breed (8–19 years) affected by EPD on hind limbs from a stud farm in Eastern Slovakia were included in the study. All horses underwent a general physical examination, followed by a thorough inspection of all four pasterns. Only long feathers were shortened with scissors (not clipped) to facilitate the application of the inoculated alginite carrier. The diagnosis of EPD was based on evidence of clinical signs (1, 4). Affected pasterns were classified into one of the three EPD forms (mild, exudative, or proliferative) according to Yu (4). All pasterns were graded for lesion severity using a standardized scoring system (12) to objectively select the worst-affected pastern. The horses had not been treated previously.

*Weissella cibaria* RIF^R^ strain stabilized on the alginite carrier was topically applied to four healthy mares on the distal part of the limb and to 12 mares affected by EPD. The same treatment was applied for 14 days; fresh treatment was applied every morning. The distal part of the limb was bandaged. Clinical signs such as erythema, signs of pruritus, pain, and the presence of skin lesions at the application site were monitored.

### 2.6 Numbers of *Weissella cibaria* RIF^R^ on the skin of the horses during and after application in the alginite carrier

Samples for determination of numbers of *W. cibaria* RIF^R^ were collected using the ESwab® collection and transport system (Copan, Murrieta, CA, USA) as four skin swabs in four directions. The samples were taken 24 h after the application (day 1), then after 7 and 14 days of application (days 7 and 14, respectively), and finally 7 days after the application period (day 21). Numbers of *W. cibaria* RIF^R^ on skin were determined by the same method as in alginite.

### 2.7 Statistical analysis

The obtained data (numbers of *W. cibaria* RIF^R^ in alginite carrier before application and on the skin after application) were analyzed using GraphPad Prism version 9.4.0 (GraphPad Software, Inc., San Diego, USA). One-way ANOVA with post-hoc Tukey’s multiple comparison test was used, and a *p*-value less than 0.05 (*p* < 0.05) was considered significant.

### 2.8 Skin swabs for amplicon sequencing

The skin was swabbed according to Ross et al. (15) with ESwab® collection and transport system before the application (day 0), after 7 and 14 days of application (days 7 and 14, respectively), and 7 days after the application period (day 21). The samples were immediately transported to the laboratory on ice and stored at −70°C.

### 2.9 Microbial community analysis

The samples were sent for DNA extraction and 16S rRNA gene amplicon library preparation and sequencing to Microsynth (Balgach, Switzerland) on dry ice. The V3–V4 region of the 16S rRNA gene was sequenced on the Illumina MiSeq platform in 2 × 250 bp reads. The obtained raw reads were already demultiplexed and did not contain non-biological sequences and biological primers. The quality of the reads was inspected using fastQC v0.11.9 (16). Due to the low sequencing depth (<1,500 reads), four samples were excluded from the dataset, which represented samples of two mares, one belonging to the control group and one belonging to the proliferative group. The mare from the proliferative group was completely removed from the dataset.

Bioinformatic processing was carried out in RStudio (R v4.0.2) using the dada2 package v1.16 (17) with default parameters. Forward and reverse reads were trimmed to 220 bp, at a maximum expected error rate of 2, allowing no Ns. Furthermore, these were merged using default parameters, and chimeras were removed with the method “consensus.” In total, 9,231 ASVs were obtained with an average number of reads per sample of 42,586 ± 10,826. ASVs were taxonomically classified using the SILVA reference database version 138 (18).

Bacterial community analyses were performed using the R packages phyloseq v1.32 (19), vegan v2.6–4 (20), and dplyr v1.1.3 (21), and plots were visualized using the ggplot2 package v3.3.5 (22). Alpha and beta diversities were calculated using the total, unfiltered community at the ASV level. ANOSIM statistics from the R package vegan were used to calculate the significance of differences between bacterial communities of healthy and EPD-affected samples (exudative and proliferative). The Mann–Whitney non-parametric test was used to assess the significance of alpha diversity indices between the groups. An unpaired t-test was used to compare the differences in the relative abundances of the genera *Weissella, Streptococcus, Staphylococcus,* and *Corynebacterium* in different experimental groups and between different days of the experiment.

## 3 Results

### 3.1 Initial microbial community of healthy and EPD-affected skin

Based on clinical signs, EPD lesions in seven mares were identified as exudative forms and five as proliferative forms. However, due to technical issues and a low number of sequencing reads, one mare was excluded from the proliferative group from the sequencing-based results. Thus, in total, 6 healthy pasterns and 11 EPD-affected pasterns were included in the bacterial community analysis based on 16S rRNA gene amplicon sequencing before the application of *W. cibaria* RIF^R^.

**Figure 1:**
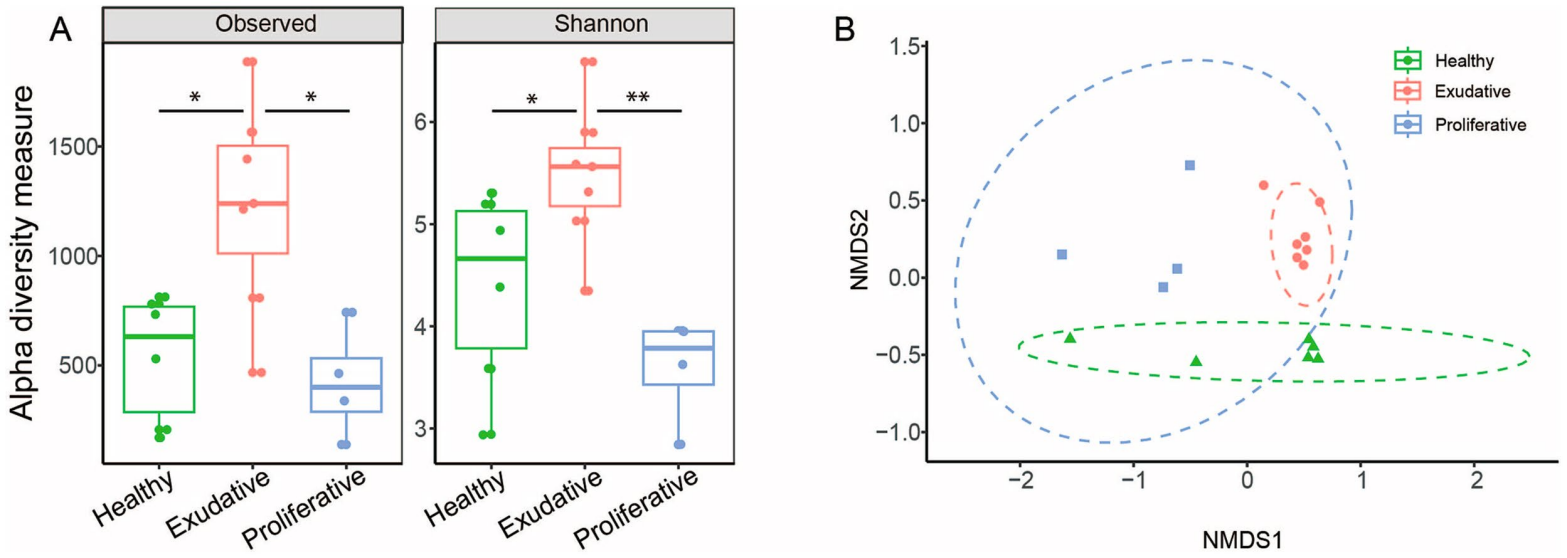
Bacterial community analysis of healthy and EPD-affected skin. **(A)** Alpha diversity indices of the 16S rRNA library at the ASV level. The dots represent individual samples. The box plot whiskers show minimum and maximum values, the bounds of the box represent the 25^th^ and 75^th^ percentile, and the median is shown at the center. **(B)** Beta diversity: non-metric multidimensional scaling plot using the Bray–Curtis dissimilarity matrix shows the relationship of individual samples in each group, at the ASV level. The ellipses represent the 95% confidence interval. Stress 0.112.

Comparison of the initial microbial community based on amplicon sequencing revealed that richness (observed diversity) and diversity (Shannon index) were surprisingly the highest in exudative lesions with relatively high in-group variability, while proliferative lesions had the lowest alpha diversity indices (Figure 1A). There was a statistically significant difference in community composition between healthy vs. exudative (*R* = 0.52, *p* = 0.003) and exudative vs. proliferative (*R* = 0.78, *p* = 0.043) skin microbiota at the ASV and genus level, but no significant differences were found in the microbiota between the healthy control group and proliferative EPD group (Figure 1B and Supplementary Table S1 online).

**Figure 2:**
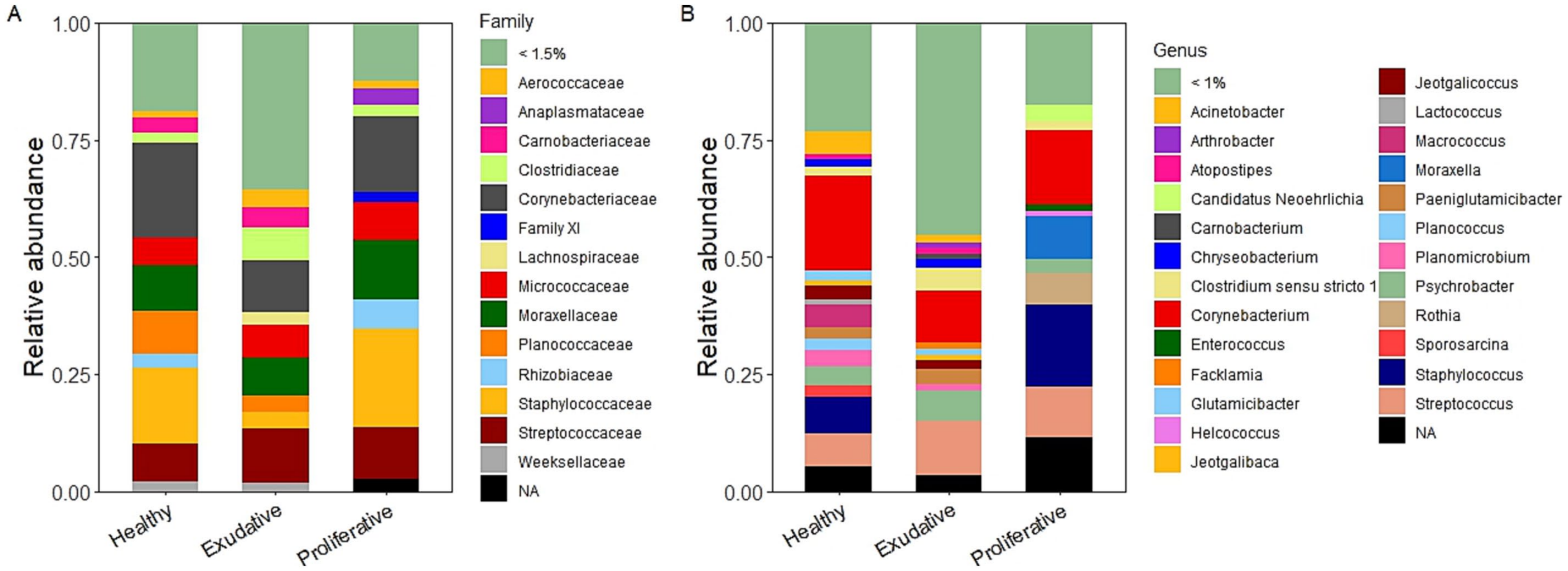
Bacterial community composition of healthy (*n* = 4) and EPD-affected skin with exudative (*n* = 7) and proliferative (*n* = 4) forms at the family **(A)** and genus **(B)** levels.

The healthy skin microbiota was dominated by the families Corynebacteriaceae (19.7 ± 15.8%) and Staphylococcaceae (15.8 ± 10.7%), followed by Moraxellaceae (9.5 ± 6.8%), Planococcaceae (9.2 ± 7.4%), Streptococcaceae (8.0 ± 1.8%), and Micrococcaceae (5.7 ± 2.7%). *Corynebacterium* was the dominating genus in the healthy group on average at 19.6 ± 15.6% but individually could represent up to half of the microbial population, followed by the genera *Staphylococcus* (7.7 ± 13.0%) and *Streptococcus* (7.0 ± 1.9%). Apart from *Staphylococcus* spp., the Staphylococcaceae family was also represented by the genera *Macrococcus* (4.6 ± 4.8%) and *Jeotgalicoccus* (2.8 ± 1.9%) in the healthy community (Figures 2A,B and Supplementary Figure S1).

The exudative and proliferative skin microbiota shared the same relative abundance of the family Streptococcaceae, represented solely by the genus *Streptococcus* (11.7 ± 4.0% and 11.5 ± 7.4%, respectively). *Streptococcus* was the dominating genus in the exudative group together with *Corynebacterium* (11.0 ± 3.8%), while *Staphylococcus* (15.6 ± 14.5%) dominated the proliferative group. *Staphylococcus* spp. represented only 0.5% of the exudative skin microbial community, which was a major difference between the EPD-affected lesion types. The family Staphylococcaceae was also represented by the genera *Jeotgalicoccus* (1.7 ± 0.9%) and *Macrococcus* (1.0 ± 1.0%) in the exudative lesions and by unidentified Staphylococcaceae (2.9 ± 4.2%) in the proliferative lesions (Supplementary Figure S1). The family Clostridiaceae, represented mainly by *Clostridium sensu stricto,* had higher abundance in the exudative group (7.0 ± 2.5%) than in the proliferative group (2.2 ± 1.9%) and was another main difference between EPD-affected skin types. The family Moraxellaceae was mainly represented by the genus *Moraxella* (10.9 ± 14.4%) in the proliferative group and by the genus *Psychrobacter* (6.7 ± 4.4%) in the exudative group (Figures 2A,B).

Based on amplicon sequencing data, *Weissella* spp. represented around or less than 0.1% of the initial community in all three groups.

### 3.2 Clinical examination

Based on clinical signs, 12 mares affected by EPD were divided into two groups: exudative (*n* = 7) and proliferative (*n* = 5). *Weissella cibaria* RIF^R^ on an alginite carrier was applied to EPD-affected lesions and healthy skin (control, *n* = 4). During and after the application of the *W. cibaria* RIF^R^ strain on alginite to the healthy skin of the distal part of the limbs, no skin reactions were observed in the horses.

In horses with an exudative form of EPD, after 1 week of application of the *W. cibaria* RIF^R^ strain stabilized on alginite, the exudative lesions dried up and were reduced by approximately half. After 2 weeks of application, the lesions were healed and not palpable. After 7 days from the end of the application, the healed exudative lesions remained impalpable. In the monitored period of 6 months after the end of the application of the *W. cibaria* RIF^R^ strain on the alginite carrier, there was no recurrence of the disease and complete healing was achieved. In horses with a proliferative form of EPD, after 1 week of application, lesions dried up and were odorless but remained palpable during the monitored period of 6 months. Changes in the bacterial community during the clinical study were monitored by cultivation (samples were taken on the 1^st^, 7^th^, 14^th^, and 21^st^ day) and by amplicon sequencing (samples were taken on day 0 and on the 7^th^, 14^th^, and 21^st^ day).

### 3.3 Cultivation-based results

**Figure 3:**
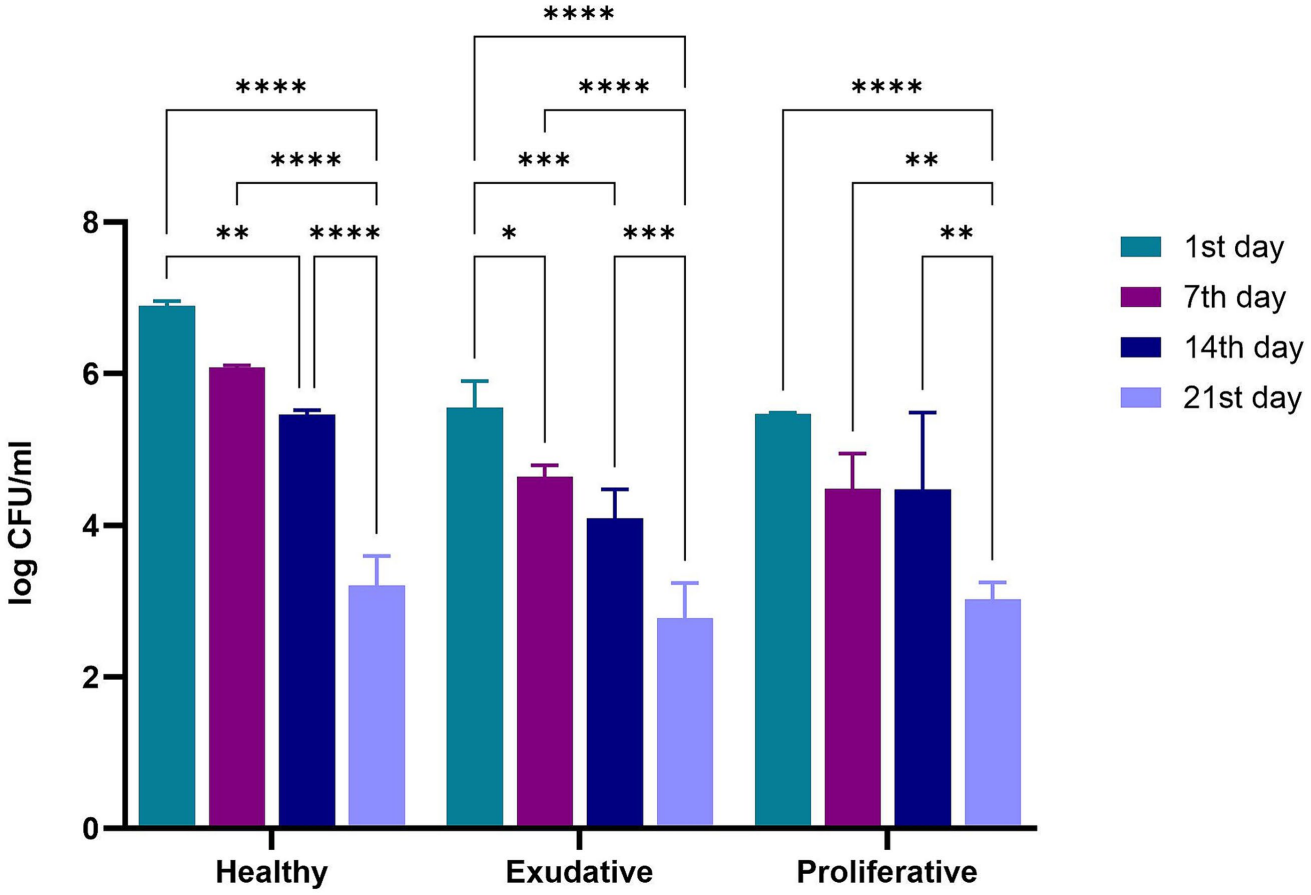
Numbers of *W. cibaria* RIF^R^ on equine skin during and after application period. Legend: *p ≤ 0.05; **p ≤ 0.01; ***p ≤ 0.001; ****p < 0.0001.

The numbers of *W. cibaria* RIF^R^ in the alginite sample at the time of application were 8.97 ± 0.38 log CFU/g for the 1^st^ week of the application period and 9.14 ± 0.34 log CFU/g for the 2nd week of the application period. The numbers of *W. cibaria* RIF^R^ on equine skin determined 24 h after the application, on the 7^th^ and 14^th^ day during the application period, and on the 21^st^ day (after 7 days without application) were the highest in the healthy group (Figure 3).

Surprisingly, the highest numbers of *W. cibaria* RIF^R^ on equine skin were on the 1^st^ day (24 h after application) in all groups. In the group of mares with exudative skin lesions, a significant decrease in the counts of *W. cibaria* RIF^R^ was observed between the 1^st^ (5.56 ± 0.35 log CFU/mL) and the 7^th^ day (4.64 ± 0.15 log CFU/mL). In all groups, the differences in the numbers of *W. cibaria* RIF^R^ were not significant on the 7^th^ vs. 14^th^ day (Supplementary Table S2) but were significant on the 1^st^ day vs. 21^st^ day. The most significant difference between the 1^st^ day and the 21^st^ day was in the group of mares with healthy skin (6.90 ± 0.07 log CFU/mL vs. 3.20 ± 0.38 log CFU/mL).

### 3.4 Community changes based on amplicon sequencing

Changes in the bacterial community during the clinical study were followed by 16S rRNA gene amplicon sequencing with samples taken before the application (day 0), on the 7^th^ and 14^th^ day of the application period, and 7 days after termination of the application (21^st^ day).

#### 3.4.1 Bacterial community during the application period of *Weissella cibaria* RIF^R^

**Figure 4:**
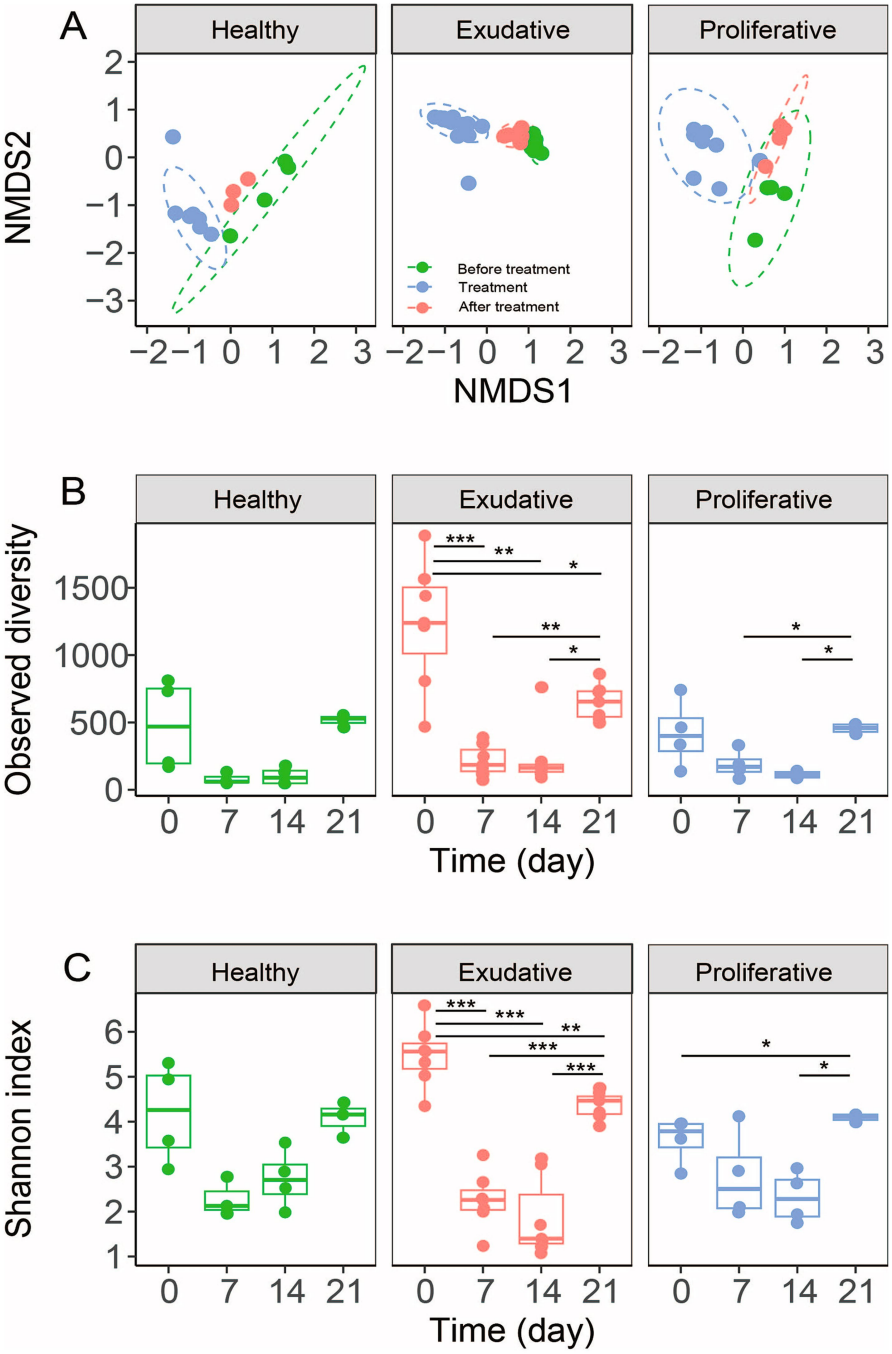
Bacterial community analysis before, during, and after the application period of *W. cibaria* RIF^R^, based on ASVs. The NMDS plot, based on Bray–Curtis dissimilarities, shows the relationship between bacterial communities **(A)**. Ellipses represent 95% confidence intervals (stress 0.146). Treatment refers to the application period, with samples taken on the 7^th^ and 14^th^ day of the application period. Alpha diversity indices are shown in panels **(B,C)**. The dots represent individual samples. The box plot whiskers represent the minimum and maximum values, the bounds of the box indicate the 25^th^ and 75^th^ percentile, and the median is shown at the center.

**Figure 5:**
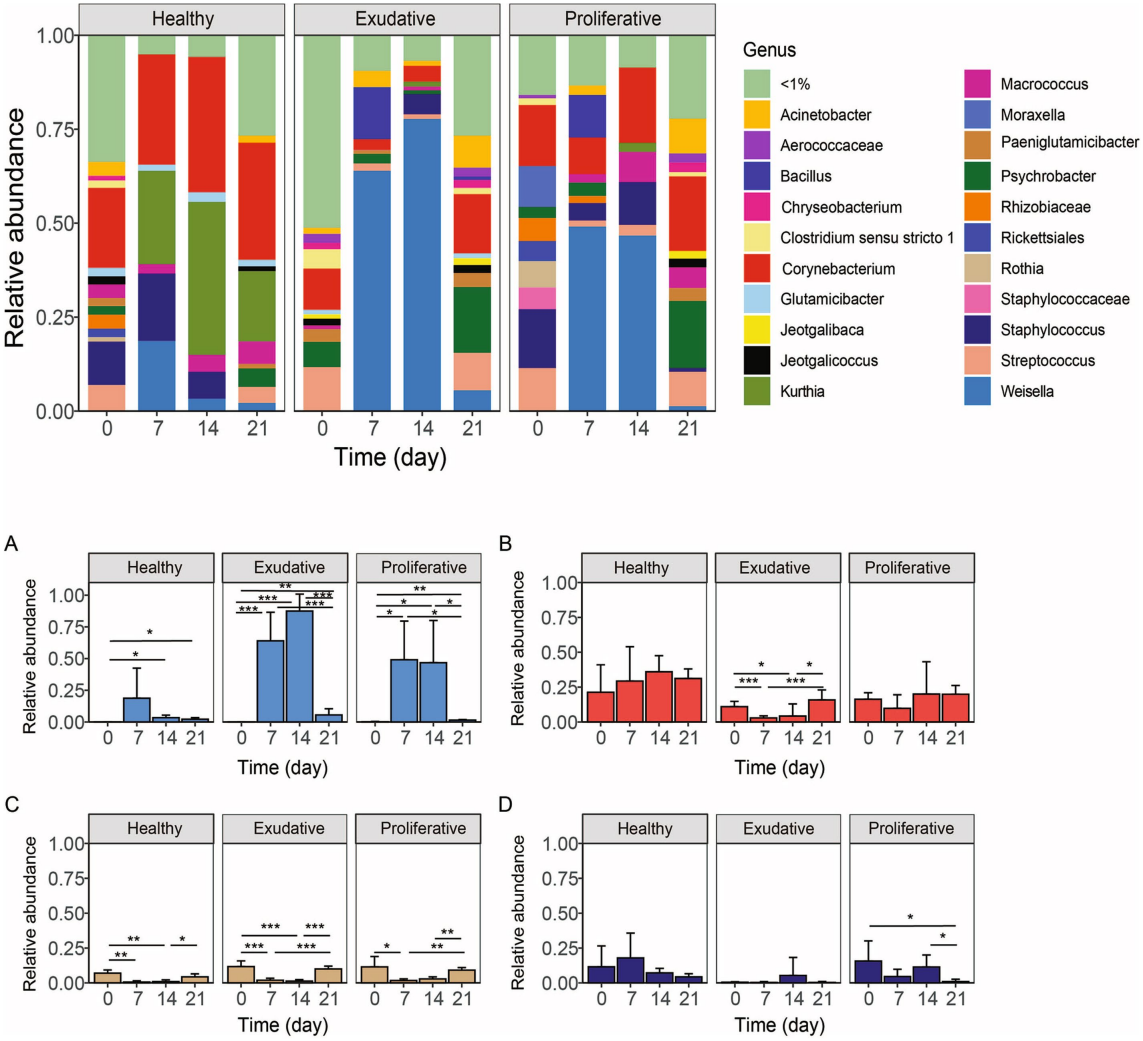
Changes in the bacterial community composition: Upper panel: Total bacterial community composition at the genus level before, during, and after the application period of *W. cibaria* RIF^R^ in the healthy (*n* = 4), exudative (*n* = 7), and proliferative (*n* = 4) skin. Lower panel: Changes in relative abundances of *Weissella* **(A)**, *Corynebacterium* **(B)**, *Streptococcus* **(C)**, and *Staphylococcus* **(D)** before, during, and after the application period of *W. cibaria* RIF^R^.

Upon application, there was a statistically significant shift in community composition in all groups, including the healthy community; however, the change was the most significant in the exudative community (*R* = 1, *p* = 0.001, Figure 4A and Table 1). During the application period (7^th^ and 14^th^ day), community composition in almost all individuals in the EPD-affected limbs was very similar (Figures 4A, 5), suggesting a successful application and good incorporation of the *W. cibaria* RIF^R^ strain into the microbial community and induction of similar changes in either the exudative or proliferative microbial community by treatment.

**Table 1:** Analysis of similarity (ANOSIM) at the ASV level, between the bacterial communities before, during, and after the application period of *W. cibaria* RIF^R^.

	Healthy	Exudative	Proliferative
0 day vs. 7^th^ day	*R* = 0.69, *p = 0.031	*R* = 1, ***p = 0.001	*R* = 0.66, *p = 0.03
7^th^ day vs. 21^st^ day	*R* = 0.52, *p* = 0.1	*R* = 0.98, ***p = 0.001	*R* = 0.68, *p = 0.036
0 day vs. 21^st^ day	*R* = 0.04, *p* = 0.7	*R* = 0.71, **p = 0.002	*R* = 0.85, *p = 0.032

On average, the genus *Weissella* represented up to 80.0 ± 13.3% of the exudative and 49.0 ± 30% of the proliferative bacterial community during the application period; however, in individual horses, it could represent up to 96% and 78% of the respective communities, signifying that its application was more effective for exudative lesions (Figures 5, 5A; this difference between exudative and proliferative was not statistically significant). In all groups, diversity and richness decreased during the application period (Figures 4B,C), and the change was the most prominent in the exudative lesions, due to the significantly higher representation of *Weissella*. In the exudative lesions, most other bacterial taxa’s relative abundance decreased along with the significantly higher representation of *Weissella* (namely the genera *Corynebacterium, Streptococcus*, *Staphylococcus, Psychrobacter*, and *Paeniglutamicibacter,* Figures 5B–D), but in the proliferative lesions, despite the dominance of *Weissella*, the genus *Corynebacterium* stayed at its initial relative abundance (Figure 5B). Relative abundance of the genus *Weissella* increased in the healthy skin microbiota as well, up to 18.0 ± 23.8%; however, it did not dominate the healthy skin bacterial community, as it did in all the EPD-affected lesions (Figure 5). Instead, an overgrowth of the genus *Kurthia* was observed in the healthy skin microbiota during the application period.

#### 3.4.2 Community 7 days after the application period of *Weissella cibaria* RIF^R^

After the application period, richness and diversity increased and were comparable in all groups with less in-group variability, contrasting the initial values mainly in the exudative group (Figures 4B,C). Analysis of beta diversity showed that the composition of the total community in the proliferative and exudative lesions was similar to the initial composition; however, differences were statistically significant in the proliferative (day 21 vs. day 0: *R* = 0.71, *p* = 0.002) and exudative (day 21 vs. day 0: *R* = 0.85, *p* = 0.032) lesions and not statistically significant in the healthy skin microbiota (day 21 vs. day 0: *R* = 0.04, *p* = 0.7; Figure 4A and Table 1).

After treatment, the genus *Weissella* remained in both exudative and proliferative lesions at a higher relative abundance compared to its initial abundances (exudative day 0 vs. 21: 0.04% ± 0.02% vs. 5.52% ± 4.89%, *p* = 0.025; proliferative day 0 vs. 21: 0.15% ± 0.25% vs. 1.33% ± 0.45%, *p* = 0.0068; Figures 5, 5A and Supplementary Table S3). The genus *Corynebacterium,* which represents a major contributor to the healthy microbiota, increased in the EPD-affected lesions in both groups (on average day 0 vs. 21: 13.6% vs. 17.8%; Figure 5B). Similarly, the genera *Psychrobacter* and *Acinetobacter* of the Moraxellaceae family increased markedly in the microbiota of both lesion types (Supplementary Table S3). The population of the genus *Streptococcus* decreased mildly in both EPD-affected groups compared to their original abundance (approximately day 0 vs. 21: 11%–12% vs. 9%–10%; Figure 5C and Supplementary Table S3). The genus *Staphylococcus* decreased significantly in the proliferative group (day 0 vs. 21: 15.6 ± 14.5% vs. 1.0 ± 1.6%; Figure 5D and Supplementary Table S3), while the genus *Clostridium* decreased in both EPD-affected lesions, with a significant reduction in the exudative group (day 0 vs. 21: 5.1 ± 2.0% vs. 1.6 ± 0.5%).

## 4 Discussion

Equine pastern dermatitis has several causes that require different diagnostic and treatment approaches. Management and treatment plans should be based on a thorough clinical assessment and identification of risk factors relevant to the individual case.

There is no treatment that works for every type of EPD, and, until now, it has remained largely empirical (23). It is essential to keep the distal parts of the limbs clean and dry and maintain good environmental hygiene.

Topical preparations are applied after gentle clipping and initial cleansing (e.g., with 2% chlorhexidine). When crusts are tightly adhered, they can be covered with a hydrogel-based dressing for 24 h under a light bandage to facilitate their removal (2). Creams or lotions containing antimicrobials (e.g., gentamicin), antifungal agents (e.g., nystatin or clotrimazole), or corticosteroids (e.g., triamcinolone, betamethasone, prednisolone, or hydrocortisone), or a combination of these are used to be applied. In dermatophyte infections, lime sulfur dips and miconazole shampoos are also recommended. Overzealous use of topical medications has to be avoided as they debilitate the skin and can cause further problems (e.g., photosensitization). Systemic antimicrobial (e.g., trimethoprim-sulfonamides) and anti-inflammatory treatment (e.g., dexamethasone, prednisolone, or pentoxifylline) is indicated when signs of pain, lameness, or elevated body temperature are present (23).

Another promising option for therapy is the use of beneficial bacteria. The study by Wilmink et al. (24) assessed the safety and systemic inflammatory effect of dressings saturated with either a standardized suspension of probiotic lactic acid bacteria or saline on the healing of traumatic distal limb wounds in horses. The probiotic-treated wounds with a complete granulation bed reached a 50% reduction in wound area in half the time of the saline-treated wounds. They also determined that the topical use of probiotics can be considered as safe as it did not cause a systemic effect.

The successful practical application of beneficial bacteria is highly dependent on the method of their application and application form, given that it is a relatively sensitive biological material. It is necessary to choose appropriate and effective forms of application to achieve an optimal probiotic effect. Alginite is a non-mineral raw material resulting from the fossilization of accumulated organic material (algae *Botryococcus braunii*) and inorganic material, mainly clay, carbonates, quartz, and the amorphous modification of silicic acid in an aqueous environment. It has high natural moisture, plasticity, relatively low weight, and high porosity (25). The method of stabilizing beneficial microorganisms by fermentation on a solid porous carrier—alginite grains, saturated with a suitable nutrient substrate and cultivation medium—is described in Styková et al. (26). The created biofilm layer and the bacterial exopolysaccharides surrounding the bacterial cells represent a barrier that isolates bacteria from the environment and ensures their resistance. Beneficial microorganisms forming a biofilm on alginite grains are alive for a long time and able to reproduce in the microenvironments of the host (26).

The genus *Weissella* colonizes various natural environments such as soil (27), plants, or freshwater lakes (28). They play an important role in the production of fermented foods (kimchi, fermented fava beans, sausages, etc.) (29, 30). They may also be present as commensals on the skin surface and in the saliva and gastrointestinal tract of humans and animals (31). Several studies have shown that *W. cibaria* has beneficial effects such as antimicrobial, antagonistic, and antioxidant effects (31, 32). Antibacterial and antibiofilm activity of the pH-neutralized cell-free supernatants of *W. cibaria* against *S. aureus* was also confirmed (14). In a study by Lim et al. (33), oral administration of *W. cibaria* reduced atopic dermatitis-like skin lesions in a murine model. A study by Yeu et al. (34) demonstrated the antimicrobial and antibiofilm properties of *W. cibaria* against *Streptococcus pyogenes*, *Streptococcus pneumoniae*, *Moraxella catarrhalis,* and *S. aureus*. Probiotic strains of *W. cibaria* also exhibit antibacterial and antibiofilm effects against *Streptococcus mutans* (35).

This is the first study to evaluate the effect of topically applied *W. cibaria* RIF^R^, stabilized on an alginite carrier, on the microbial community of EPD-affected skin lesions. EPD is associated with changes in the microbial composition of the skin. Several studies have confirmed that alpha diversity is reduced in EPD, with more pronounced changes and reduced diversity of the microbiota occurring in more severe lesions (12, 13, 36). In our study, a significantly reduced microbial diversity was found only in the proliferative (more severe) skin lesions, while in the exudative (milder) lesions, the diversity was surprisingly the highest among the three groups compared, but also with the highest in-group variability (Figure 1A). Despite the average alpha diversity being the highest in the exudative group, beta diversity showed that the lesions in individual horses shared a very similar bacterial composition, mainly from the dominating phylogroups (Figure 1B). Beta-diversity analysis also showed that all three groups had distinct microbial compositions.

Comparing the composition of the initial (pre-application) microbiota in the healthy and EPD-affected lesions (Figure 2B), the genus *Streptococcus* was considerably more abundant in both EPD groups than in the healthy group. *Staphylococcus* spp. dominated only in the proliferative group, and its abundance in the exudative group was consistently low in all the individual horses, representing 0.08%–0.98%. Previous studies, analyzing the microbial composition of EPD-affected lesions, also showed an increased abundance of the genera *Streptococcus* (13) and *Staphylococcus* (12, 13) in moderate to severe (*Streptococcus* spp.) or only in severely affected lesions (*Staphylococcus* spp.). A significantly higher prevalence of *S. aureus* with specific virulence factors was found on skin affected by EPD (36). Some authors suggested the contribution of increased moisture and opportunistic pathogens such as *S. aureus* to the development and course of EPD (8, 23). The application of *W. cibaria* RIF^R^ was associated with positive changes in the microbiota during and after the application period. Most importantly, the application caused a statistically significant decrease in the genus *Staphylococcus* in the proliferative group after the application period (Figure 5D and Supplementary Table S3). The application also led to a decrease in *Streptococcus* spp. in both EPD groups; however, this was not statistically significant.

The genus *Corynebacterium* was a major phylogroup in all the groups and dominated the healthy and proliferative microbiota. The application of *W. cibaria* RIF^R^ in the healthy and proliferative groups was associated with the increase of *Corynebacterium* during the application period and with an increase of this genus in all the groups after termination of the application (Figure 5B and Supplementary Table S3). In previous studies, a higher relative abundance of the family Corynebacteriaceae (12) or the genus *Corynebacterium* (13) was associated rather with EPD lesions or with their more severe forms. *Corynebacterium* represents a dominant member of the human and equine healthy skin microbiome (37, 38). Some *Corynebacterium* spp. are opportunistic pathogens and coexist among healthy skin microbiota, while other species are toxigenic and classified as serious and potentially life-threatening pathogens (39). *Corynebacterium pseudotuberculosis* causes infections that take different forms in horses, including external or internal abscesses or infection of the limbs also called ulcerative lymphangitis (40, 41). It has been shown that *Corynebacterium diphtheriae* and *Corynebacterium ulcerans* can readily spread among horses within the same stable, complicating pastern dermatitis infections (42).

Although no statistically significant difference was detected between the initial microbial community of the healthy and proliferative groups in the present study, there was a considerably higher representation of the genera *Staphylococcus, Streptococcus*, *Moraxella*, and *Rothia* in the proliferative group. The role of the genera *Moraxella* and *Rothia* in EPD is currently unknown and needs further studies. In a previous study, *Moraxella* had a higher relative abundance in the moderate-to-severely affected group (13), similar to our findings, while in another study, the family Moraxellaceae had the highest relative abundance in the control pasterns (12). The genus *Rothia* was found to be present in higher relative abundance in the non-lesional or mildly affected group than in the moderate to severely affected EPD group (13). In our study, the application of *W. cibaria* RIF^R^ significantly decreased the relative abundance of both *Moraxella* and *Rothia* genera in the proliferative group, similar to its effect on *Staphylococcus* spp. (Supplementary Table S3). The family Moraxellaceae includes commensals of the mucous membranes of humans and other mammals, but some *Moraxella* spp. are opportunistic pathogens (43). For example, *Moraxella osloensis* is part of the human skin microbiome and is often involved in human infectious diseases (44). It is also considered that *Moraxella* spp. can be pathogenic with the potential to cause deep soft tissue infections (45). Representatives of the genus *Rothia* commonly colonize the upper respiratory tract and oral cavity of humans and animals (46). Some *Rothia* spp. are considered an opportunistic pathogen, particularly in immunocompromised patients, while *Rothia kristinae* is commonly found on human skin (47). In the exudative group, there was a higher representation of *Psychrobacter* and *Clostridium sensu stricto* 1 compared to the proliferative and healthy groups. In previous studies, the genus *Psychrobacter* was found to represent the top10 most abundant genera either in the cutaneous microbiota of healthy horses (37) or in all forms of EPD-affected skin (non-lesional, mild, moderate, or severe; (13). However, it had a higher percentage representation in the moderate-to-severely affected EPD group. *Clostridium sensu stricto* was not described in the mentioned studies. In general, *Clostridium sensu stricto* is considered the true *Clostridium* genus, which includes important pathogens for humans and animals and industrially relevant microorganisms (48).

In a healthy group, during the application period of *W. cibaria* RIF^R^, overgrowth of the genus *Kurthia* was observed. The little-known genus *Kurthia* includes three species: *K. zopfii*, *K. gibsonii,* and *K. sibirica*. As the type species of this genus, *K. zopfii* has been isolated from intestinal contents, fecal material, meats, meat products, milk, water, and air (49). Due to its strictly aerobic nature, it was long assumed to be non-pathogenic. However, it was later reported that *K. gibsonii* can play the role of an opportunistic pathogen in poultry, although it does not cause primary infections (50, 51).

The data presented in this study suggest that the *W. cibaria* RIF^R^ was incorporated into the exudative lesions with a higher efficiency than into the proliferative lesions (Figures 5, 5A), which corresponds to the complete healing of the exudative lesions. Since *Weissella* was incorporated into the healthy skin microbiota as well, perhaps the application on healthy skin could serve as prevention against EPD relapse; however, our data are not sufficient for a clear statement. Based on previous (34, 35) and our results, we can assume that *W. cibaria* has an antibacterial effect against streptococci. Moreover, in our study, it affected representatives of the genera *Moraxella* and *Rothia*, as their relative representation dropped below 0.05% during and after the application period of *W. cibaria* RIF^R^.

After the termination of the application of *W. cibaria* RIF^R^, the relative abundance of the genus *Weissella* remained higher in all groups than in its initial abundance (Figure 5A). Furthermore, the richness and diversity of microbiota increased compared to the initial microbial community and were comparable in all groups (Figures 4B,C). Similar results were obtained in the study by Kamus et al. (52), where after the completion of healing of experimentally induced wounds, the equine skin microbiota had a similar composition to the controls with higher diversity.

In this study, healthy horses served as the controls. Additionally, the microbiome of EPD-affected horses was analyzed before application, which served as another level of control. However, horses that would receive treatment with alginite only were not included. This may be regarded as a limitation of the current study.

In conclusion, the application of *W. cibaria* RIF^R^ strain stabilized on alginite affected the microbial composition of EPD-affected lesions. Cultivation and 16S rRNA amplicon sequencing confirmed its ability to survive on equine skin and influence the pathogenic microbiota. At the time of application, a decrease in representatives of the genera *Streptococcus*, *Moraxella*, *Rothia*, and others was observed, and after the application period, a significant reduction in *Staphylococcus* and *Clostridium* numbers was noted. Statistically significant changes in the microbiota composition were mainly observed in horses with the exudative forms of EPD. In addition to microbial changes, skin lesions were no longer palpable after 2-week treatment in the exudative group, with no recurrence during 6-month monitoring following the end of the application. Based on these results, we conclude that a topically applied *W. cibaria* RIF^R^ stabilized on alginite may have potential as a therapeutic option for EPD, particularly in patients with exudative lesions. However, to establish a direct link between our clinical observations and the application of *W. cibaria* RIF^R^, further studies using additional control groups are necessary.

## Patents

One patent resulted from the work reported in this study. The patent titled: Microbial strain *Weissella cibaria* 4/8 D37 CCM 9015 bacterial culture, cell-free supernatant of the strain, and the pharmaceutical composition containing this strain. A patent is registered in the Industrial Property Office of the Slovak Republic under number SK 289178 (27 March 2024, Banská Bystrica).

## Data Availability

The datasets presented in this study can be found in online repositories. The names of the repository/repositories and accession number(s) can be found below: https://www.ncbi.nlm.nih.gov/genbank/, PRJNA1068551.
