# Supplementary material for: Microbiological effect of topically applied Weissella cibaria on equine pastern dermatitis
Source: Front Vet Sci. 2025 Jan 6;11:1493756. doi: 10.3389/fvets.2024.1493756 (PMC11743981; doi:10.3389/fvets.2024.1493756)
Supplement: Supplementary file 1 [file Table_1.docx]

**Supplementary Table S1.** Analysis of similarity (ANOSIM) at the ASV level between healthy, exudative and proliferative bacterial communities

before the application of *Weissella cibaria* RIF^R^

| **Healthy vs. exudative** | **Healthy vs. proliferative** | **Exudative vs. proliferative** |
| --- | --- | --- |
| R=0.52, ***p*=0.003 | R=0.23, ns *p*=0.134 | R=0.78, **p*=0.043 |


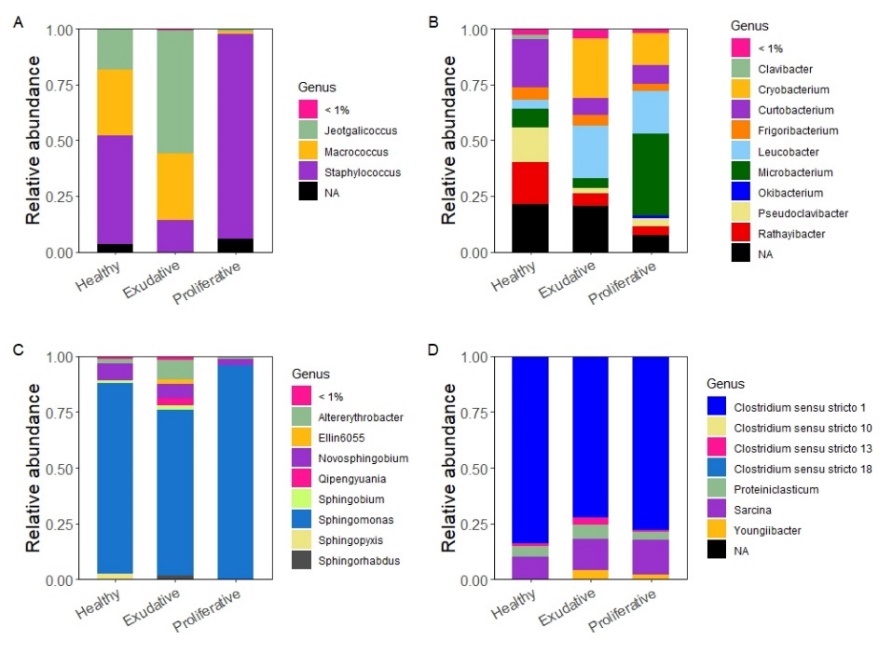


**Supplementary Figure S1.** Comparison of bacterial community composition within the Staphylococcaceae (**A**), Micrococacceae (**B**), Sphingobacteriaceae (**C**) and Clostridiaceae (**D**) families in the healthy skin and EPD-affected exudative and proliferative lesions before the application of *W. cibaria* RIF^R^

**Supplementary Table S2.** Statistical analysis of determined numbers of *W. cibaria* RIF^R^ in skin swabs based on cultivation

|  | **Healthy** | **Exudative** | **Proliferative** |
| --- | --- | --- | --- |
| 1^st^ day vs. 7^th^ day | ns *p*=0.1782 | **p*=0.0145 | ns *p*=0.0786 |
| 1^st^ day vs. 14^th^ day | ***p*=0.0064 | ****p*=0.0002 | ns *p*=0.0747 |
| 1^st^ day vs. 21^st^ day | *****p*<0.0001 | *****p*<0.0001 | *****p*<0.0001 |
| 7^th^ day vs. 14^th^ day | ns *p*=0.3908 | ns *p*=0.2165 | ns *p*>0.9999 |
| 7^th^ day vs. 21^st^ day | *****p*<0.0001 | *****p*<0.0001 | ***p*=0.0055 |
| 14^th^ day vs. 21^st^ day | *****p*<0.0001 | ****p*=0.0005 | ***p*=0.0059 |

**Supplementary Table S3.** Relative abundance (average ±SD) of the most abundant taxonomic groups (family and genus level) before, during and after the termination of the application of *W. cibaria* RIF^R^

|  | Healthy control | | | Exudative | | | Proliferative | | |
| --- | --- | --- | --- | --- | --- | --- | --- | --- | --- |
| Taxonomic group | 0 day  (n=4) | 14^th^ day  (n=3) | 21^st^ day  (n=3) | 0 day  (n=7) | 14^th^ day  (n=7) | 21^st^ day  (n=7) | 0 day  (n=4) | 14^th^ day  (n=4) | 21^st^ day  (n=4) |
| *Weissella* | 0.05±0.02% | 3.4±2.0% | 2.2±1.3% | 0.04±0.02% | 77.7±30.4% | 5.5±4.9% | 0.2±0.3% | 46.7±33.3% | 1.3±0.5% |
| Corynebacteriaceae | 21.4±19.9% | 36.1±11.6% | 31.4±21.8 | 11.0±3.8% | 4.2±8.7% | 15.8±7.2% | 16.4±4.7% | 20.4±23.8% | 20.4±23.8% |
| *Corynebacterium* | 21.3 ±19.7% | 36±11.5% | 31.1±7% | 11.0±3.8% | 4.2±8.7% | 15.8±7.2% | 16.3±4.7% | 20.0±23.2 | 19.3±6.3% |
| Staphylococcaceae | 18.0±11.8% | 12.0±4.7% | 12.8±2.6% | 3.3±1.1% | 6.8±15.6% | 2.6±1.6% | 19.5±13% | 20.2±22.3% | 9.3±2.3% |
| *Staphylococcus* | 11.5±15% | 7.1 ±3.3 % | 4.3±2.3% | 0.5±0.3% | 5.3±12.9% | 0.3±0.7% | 15.6±14.5% | 11.4±8.6% | 1.0±1.6% |
| *Macrococcus* | 3.6±3.1% | 4.5 ±1.7% | 6.0±2.9% | 1.0±1.0% | 0.98±2.0% | 0.05±0.04% | 0.5±0.7% | 8.0±15.2% | 5.6±10.7% |
| *Jeotgalicoccus* | 2.1±1.8% | 0.1 ±0.1% | 1.2±0.9% | 1.8±0.9% | 0.2±0.3% | 2.2±0.9% | 0.4±0.3% | 0.1±0.1% | 2.3±0.6% |
| Moraxellaceae | 6.7±4.2% | 0.3±0.4% | 6.8±2.1% | 8.5±4.3% | 2.4±3.0% | 26.1±7.9% | 14.1±12.8% | 0.8±1.1% | 27.2±11.7% |
| *Psychrobacter* | 2.3±1.8% | 0.2±0.2% | 4.9±3% | 6.7±4.7% | 1.0±1.2% | 17.5±4.8% | 2.9±3.0% | 0.4±0.5% | 17.9±8.8% |
| *Acinetobacter* | 3.7±4.0% | 0.1 ± 0.2% | 1.9±1.1% | 1.6±0.7% | 1.4±1.9% | 8.6±3.7% | 0.3±0.3% | 0.4±0.5% | 9.3±3.1% |
| *Moraxella* | 0.4±0.8% | 0.0 ± 0.0 | 0.0±0.0 | 0.0±0.0 | 0.0±0.0 | 0.0±0.0 | 10.9±14.4% | 0.0±0.1% | 0.0±0.1% |
| Planococcaceae | 6.9±7.7% | 40.8±18.6% | 20.6±6.5% | 3.5±2.3% | 2.1±2.9% | 2.2±1.0% | 0.6±0.6% | 2.9±5.6% | 1.5±0.7% |
| *Kurthia* | 0.1±0.1% | 40.7±18.6% | 18.7±11.5% | 0.0±0.0% | 1.34±2.5% | 0.0±0.0% | 0.0±0.0% | 2.5±4.9% | 0.1±0.2% |
| Streptococcaceae | 8.6±1.9% | 1.2±1.3% | 4.3 ±3.0 | 11.7±4.1% | 1.3±1.1% | 10.0±2.0% | 11.6±7.5% | 2.8±1.5% | 9.2±1.8% |
| *Streptococcus* | 7.0±2.3% | 0.9±1.3% | 4.3±2.2 | 11.7±4.1% | 1.3±1.1% | 10.0±2.0% | 11.5±7.4% | 2.8±1.5% | 9.2±1.8% |
| Micrococcaceae | 6.1±3.4% | 2.7±2.7% | 3.5±0.7% | 6.7±1.7% | 0.7±0.6% | 6.0±0.7% | 8.5±7.5% | 0.3±0.2% | 6.0±1.7% |
| *Rothia* | 1.2±2.4% | 0.0±0.0 | 0.0±0.0% | 0.1±0.1% | 0.0±0.0% | 0.0±0.0% | 7.0±7.8% | 0.0±0.0% | 0.0±0.0% |
| *Paeniglutamicibacter* | 2.1±2.7% | 0.1±0.1% | 1.2±0.6% | 3.4±1.1% | 0.3±0.3% | 3.7±0.5% | 0.6±0.4% | 0.0±0.1% | 3.4±1.0% |
| *Glutamicibacter* | 2.3±1.6% | 2.5±2.7% | 1.8±0.7% | 1.2±0.5% | 0.1±0.2% | 1.3±0.2% | 0.3±0.2% | 0.0±0.0% | 1.6±0.7% |
| *Clostridium sensu stricto 1* | 2.0±1.1% | 0.0±0.0 | 0.8±0.3% | 5.1±2.0% | 0.2±0.3% | 1.6±0.5% | 1.8±1.6% | 0.0±0.0% | 1.1±0.1% |
